# Paralogous HOX13 Genes in Human Cancers

**DOI:** 10.3390/cancers11050699

**Published:** 2019-05-20

**Authors:** Gerardo Botti, Clemente Cillo, Rossella De Cecio, Maria Gabriella Malzone, Monica Cantile

**Affiliations:** 1 Scientific Direction, Istituto Nazionale Tumori IRCCS Fondazione G. Pascale, 80131 Naples, Italy; g.botti@istitutotumori.na.it; 2 Department of Clinical Medicine and Surgery, School of Medicine, University of Naples Federico II, 80131 Naples, Italy; clecillo@unina.it; 3 Pathology Unit, Istituto Nazionale Tumori IRCCS Fondazione G. Pascale, 80131 Naples, Italy; r.dececio@istitutotumori.na.it; 4 Pathological Anatomy Laboratory, Casa di Cura Maria Rosaria, 80045 Pompei, Italy; gabriellamalzone@gmail.com

**Keywords:** HOX gene network, paralogous HOX 13 genes, tumor diseases

## Abstract

Hox genes (HOX in humans), an evolutionary preserved gene family, are key determinants of embryonic development and cell memory gene program. Hox genes are organized in four clusters on four chromosomal loci aligned in 13 paralogous groups based on sequence homology (Hox gene network). During development Hox genes are transcribed, according to the rule of “spatio-temporal collinearity”, with early regulators of anterior body regions located at the 3’ end of each Hox cluster and the later regulators of posterior body regions placed at the distal 5’ end. The onset of 3’ Hox gene activation is determined by Wingless-type MMTV integration site family (Wnt) signaling, whereas 5’ Hox activation is due to paralogous group 13 genes, which act as posterior-inhibitors of more anterior Hox proteins (posterior prevalence). Deregulation of HOX genes is associated with developmental abnormalities and different human diseases. Paralogous HOX13 genes (HOX A13, HOX B13, HOX C13 and HOX D13) also play a relevant role in tumor development and progression. In this review, we will discuss the role of paralogous HOX13 genes regarding their regulatory mechanisms during carcinogenesis and tumor progression and their use as biomarkers for cancer diagnosis and treatment.

## 1. Introduction

Homeobox genes, a superfamily of transcription factors, are considered as key determinants of embryonic development and body plan organization [1]. Among them, Hox genes (HOX in humans) represent a conserved gene family controlling antero-posterior axis and dorsum-ventral organization during development [2]. HOX genes are organized into the HOX gene network: four chromosomal loci (HOXA chr.7p15, HOXB chr.17q21, HOXC chr.12q13, HOXD chr.2q31-33) [3] for a total of 39 genes aligned into 13 vertical paralogous groups on the basis of sequence similarity and position in the loci [4]. The Hox gene network organization has evolved from a single ancestral proto-Hox gene through replications and transpositions [5].

The Hox gene network cooperates with the Polycomb and Trithorax gene families to mediate the cell memory program, with the Hox genes regulating mRNA transcription and Polycomb and Trithorax genes blocking or inducing DNA-chromatin interaction, respectively [6]. However, while Polycomb and Trithorax gene family members are dispersed throughout the genome [7], the HOX genes exists in tightly regulated clusters that form the largest physically and functionally identifiable network in the human genome as reported by Lander et al. [8]. 

During mammalian development, Hox genes begin to be expressed at gastrulation, controlling the identity of body regions, along the antero-posterior axis, according to the rules of spatio-temporal collinearity [9]. Hox genes are activated at the 3’ end loci in response to Wnt3 signals. Subsequent induction of Hox genes located at the 5’ end, following the same dynamics, generates the antero-posterior elongation. The activation of the 5’ end HOX genes is controlled by paralogous group 13 HOX genes, which play a crucial role in the retro-inhibition of the functions of more anterior Hox proteins, limiting axial elongation. This negative-dominant effect, called “posterior prevalence”, is due to the prevalent role of posterior Hox proteins on more anterior genes of the Hox network. The whole process induces the translation from temporal information to a series of spatial coordinates (spatio-temporal collinearity) [10]. Studies on the expression of the HOX network have shown unique patterns (HOX profiles) in different tissues and organs. These observations support the conclusion that: (i) the HOX gene network expression model for each organ is the sum of the different HOX profiles of the organ resident cells; (ii) the organ specific HOX profiles contribute to the anatomical organization of the organs and to the appropriate positioning of each organ along the antero-posterior axis of the body [11].

The post-genomic era has identified the HOX network as one of the richest areas of the genome for its content of non-coding RNAs (ncRNAs), including both microRNAs (miRNAs) and long-non-coding-RNAs (lncRNAs) [12]. Approximately 240 ncRNAs have been identified within the HOX network and less than 10% have been functionally characterized so far. Among them, key players are represented by HOTTIP, for its localization (adjacent to HOXA13)/interaction with posterior locus A HOX genes [13,14] and HOTAIR for its property to regulate *in trans* HOXD locus [15]. The understanding of the HOX network organization and activity has been profoundly boosted by the identification of the non-coding RNA world. 

The comparison of HOX profiles in normal and neoplastic tissues has allowed the identification of HOX genes dysregulated in both tumor- and tissue-specific manner [16,17]. Their deregulation during tumor progression is associated with the tumor heterogeneity, epithelial mesenchymal transition and metastasis [18,19]. Since HOX genes also control important metabolic processes, the identification of unique HOX profiles associated to primary tumors has provided an important glimpse on the deregulation of primary metabolic processes contributing to specific tumor phenotypes and, more generally, to the biology of cancer cells [20,21]. Although the entire HOX network plays a central role in cancer development and progression, the most posterior genes of the network, HOX13 paralogues (HOXA13, HOXB13, HOXC13 and HOXD13), specifically, are crucial in modulating these processes, in cooperation with co-localizing lncRNAs. 

During development, all HOX13 paralogues are involved in mediating the transition from the early to the late-distal limb program, controlling the spatial expression patterns of target genes [22] and in mediating gut and urogenital system formation [23,24,25]. However, many of them are still active in adult human organs and tissues [26] and frequently deregulated in human cancers [26,27,28,29]. 

Due to the crucial role played by paralogous HOX13 genes on the HOX gene network organization during embryonic development, as well as in tumor progression, in this review we will discuss: (i) their physiological and pathological regulatory mechanisms during carcinogenesis; (ii) their ability to modulate tumor progression also through the interaction with co-localized ncRNAs; (iii) their potential applications as biomarkers for cancer diagnosis and treatment. 

## 2. HOXA13

During normal development, HOXA13 plays a leading role in the creation of posterior structures of the body, specifically in limb, gut and urogenital system development [22,24,30]. During abnormal development, modifications of HOXA13 homeoprotein have been associated with “Hand-foot-genital” syndrome [31].

HOXA13 deregulation in cancer has been recently validated by a meta-analysis study. In 844 tumor patient biopsies, enrolled in nine different studies, the aberrant expression of HOXA13 is significantly associated with poor histological grade, TNM stage and overall survival, suggesting that HOXA13 is a potentially valuable biomarker of poor prognosis and potential therapeutic target for human tumors. [32]. 

HOXA13 deregulation has been strongly associated with urological cancers (bladder and prostate). High levels of HOXA13 homeoprotein have been found in bladder cancer tissues and positively correlated with lymph nodes metastases, TNM stage, pathological grade and patient survival [33]. Moreover, since circulating bladder tumor cells can be carried in the urine, HOXA13 level has been also detected in this biological fluid. cDNA microarray analysis has highlighted that co-expression of HOXA13 and BLCA-4 could be able to discriminate low versus high grade bladder tumors [34]. These data have been then confirmed by STRING database screening which, in turn, has allowed the identification of a four-gene model, including IGF-1, hTERT, BLCA-4 and HOXA13 to stratify bladder cancers at different stages [35]. Aberrant HOXA13 expression has been reported in prostate cancer (PC), where nuclear HOXA13 expression is strongly associated with histological grade and Gleason score. Moreover, induced HOXA13 expression in PC cell models promoted cell proliferation, migration, invasion and inhibition of apoptosis [36]. HOXA13 is able to frequently form a fusion gene with nucleoporin NUP98, named NUP98-HOXA13, playing a key role in acute myeloid leukemia (AML) [37,38]. A chromosomal translocation between an upstream HOXA13 region and a downstream region of the BCL11B/CTIP2 locus has been described in T-cell acute lymphoblastic leukemia (T-ALL), resulting in a HOXA13 gene hyper-expression [39]. HOXA13 is strongly up-regulated in gastric cancer (GC) tissues compared to normal adjacent mucosa. HOXA13 aberrant expression correlates with GC tumor stage, histological differentiation and survival of the patient [40]. HOXA13 is also hyper-expressed in gastric stem cells [41] and its knockdown, in GC cell model, modulates epithelial-mesenchymal-transition (EMT) reducing cell invasion features [42]. A recent study has highlighted that HOXA13 over-expression significantly increases cadherin17 (CDH17) gene expression in GC cells and tissues. The simultaneous knockdown of both genes leads to a reduction of cell proliferation and invasion and promotes apoptosis in GC cell model [43]. Han and collaborators have lately shown that HOXA13 is strongly related to 5-fluorouracil (5-FU) resistance in GC patients. HOXA13 might confer resistance to GC cells by a p53-dependent pathway [44]. HOXA13 deregulation has been further associated with Disease Free Survival (DFS) in esophageal squamous cell carcinoma (ESCC) [45,46]. The knockdown of HOXA13 in ESCC cell model leads to a reduced number of colonies in vitro and tumor growth in nude mice [46]. The coordinated expression of HOXA13, ANXA2 and SOD2 strongly predicts poor prognosis in ESCC [47]. HOXA13 is also involved in the modulation of EMT in ESCC cells [48]. In ESCC patients treated with neoadjuvant chemotherapy, HOXA13 expression is associated with the worst tumor regression grade. The knockdown of HOXA13, in ESCC cells, increases cis-platinum-induced apoptosis, suggesting an essential role of HOXA13 in drug-resistance acquisition [48]. In a further investigation, an opposite trend of HOXA13 expression has been detected in oral squamous cell carcinoma (OSCC): a prevalent HOXA13 expression, in the superficial side of the lesions, is significantly associated to a better prognosis of OSCC patients [27].

There is also growing evidence that HOXA13 has a role in liver cancer. HOXA13 is over-expressed in primary hepatocellular carcinoma (HCC) and is strongly associated with hepatitis B and C virus infection. In addition, its expression has been detected in HCC cell lines originating from liver stem-like cells, suggesting the HOXA13 role in the differentiation and tumor evolution of hepatic stem cells [49]. The profile of the whole HOX network in a large cohort of paired liver biopsies, HCC versus their non-neoplastic counterparts, has identified the locus A HOX gene as the most dysregulated locus among the HOX loci and HOXA13 is systematically over-expressed in HCCs versus normal/non-neoplastic livers. The study has demonstrated that HCC samples with high HOXA13 expression manifest the dysregulation of a gene set associated to poor prognosis, according to HCC transcriptome classification. Furthermore, HOXA13 homeoprotein physically interacts with the cap-binding protein eIF4E, deregulated in HCC [50]. HOXA13 expression in HCC patients is also strongly correlated with the expression of angiogenic markers, such as VEGF, microvessels density and alpha-fetoprotein (AFP) serum levels. In addition, serum HOXA13 levels have been detected in 90 HCC patients suggesting that its circulating level could be used for early HCC diagnoses and prediction of the outcomes [51]. In HCC in vitro model, HOXA13 further correlates with poor differentiated HCC modulating sorafenib response [52]. The deregulation of HOXA13 has been also described in lung cancer. The expression data of HOXA13 have been collected from different databases, highlighting its aberrant expression mainly in lung adenocarcinoma progression [53]. In addition, Kang and collaborators have described a frequent gain of copies number on the short arm of chromosome 7 containing the whole locus HOXA, suggesting its critical role in lung adenocarcinoma evolution [54]. HOXA13 deregulation has been sporadically associated to other cancer phenotypes, such as ovarian cancer associated with poor clinical outcome [55], in glioma associated with tumor progression thought Wnt and TG-Beta pathways modulation [56] and thyroid cancers where HOXA13 nuclear expression is associated with different histotypes [29].

In recent studies, the aberrant role of HOXA13 in cancer is frequently associated with HOTTIP expression, suggesting that their interaction is strongly related to the modulation of tumor evolution and progression. LncRNA HOTTIP (HOX transcript at distal tip) has been functionally characterized in 2011 by Chang [56]. HOTTIP is located at the 5’ end of the locus HOXA on chromosome 7p15, adjacent to HOXA13. According to its genomic localization, HOTTIP is active, from development to adulthood, in lumbar-sacral body regions [57]. Through interaction with the activator Trithorax complex WDR5/MLL(H3K4me3), HOTTIP is able to promote the activation of a block at 5’ end of HOXA locus genes, from HOXA13 to HOXA9 [57]. In the mouse model, HOTTIP knock-out generates alterations very similar to HOXA11 and HOXA13 inactivation, supporting its role in the functional control of the locus HOXA lumbar-sacral region [57]. Based on the physical and functional interaction between HOTTIP and HOXA13, as well as the role played by HOXA13 in HCC, the role of HOTTIP in HCC has been also investigated accordingly. HOTTIP is significantly over-expressed in HCCs versus normal liver tissues, likewise HOXA13 [14]. The levels of HOXA13 and HOTTIP are able to predict HCC prognosis being associated to metastasis status, clinical outcome and patients’ survival [14]. LncRNA HOTTIP has been described as over-expressed in pancreatic cancers promoting tumor progression and EMT. HOTTIP is also highly active in pancreatic stem cells affecting stem cell factors (LIN28, NANOG, OCT4 and SOX2) and markers (ALDH1, CD44 and CD133) through a mechanism involving HOTTIP/WDR5/HOXA9/Wnt/beta-catenin axis. [58]. HOXA13-HOTTIP interaction has been also documented in GC progression: in GC cell model the knockdown of HOTTIP is strongly related to poor differentiated GC, TNM stage and lymph nodes metastasis [59]. HOXA13 is able to trans-activate IGFBP3 promoter in GC cells promoting their oncogenic potential. Its knockdown leads to a deregulation of both HOTTIP and IGFBP3, suggesting that HOXA13/HOTTIP/IGFBP3 cascade is strongly involved in GC carcinogenesis [60]. The main role of HOTTIP and HOXA13 in prostate cancer has been underlined by the recent finding that PC risk elements are mainly related to HOXA13 and HOTTIP expressions, but not to other HOXA locus genes [61]. In addition, the knockdown of HOXA13 and HOTTIP in PC cell models leads to a deregulation of different cell cycles and cell growth pathways [62]. The combined aberrant expression of HOXA13/HOTTIP related to the promotion of cell proliferation and in vivo/in vitro metastases, has been also reported in ESCC cells [63] and in non-small-cell-lung cancer (NSCLC) [64].

The coordinated dysregulation of HOTTIP and HOXA13 in different tumor types supports their strong interaction and involvement in general mechanisms of neoplastic transformation, regardless of specific tumor phenotypes. 

## 3. HOXB13

HOXB13 plays an important role in dermis development, as well as being involved with other HOX13 paralogues in the formation and organization of posterior body structures [65,66] and in the modulation of prostate differentiation and function [67] by regulating the response to androgens [68].

The role of HOXB13 in human cancer has been mostly associated to breast cancer (BC) and PC. Initial analyses on BC have highlighted the over-expression of HOXB13 in tumor tissues compared to adjacent non-neoplastic mammary gland [69]. Later on, Ma and collaborators, through a gene expression profiling study on a case series of estrogen receptor positive (ER+) BC patients treated with adjuvant tamoxifen (TAM), have identified two genes differentially over-expressed, HOXB13 in TAM recurrences and IL17BR in non-recurrences patients, suggesting the existence of a HOXB13/IL17BR ratio to predict TAM response in BC patients [69]. Subsequent studies have validated the two-gene expression ratio for therapeutic stratification of ER+BC patients [70,71,72]. High HOXB13/IL17BR expression also represents a strong independent prognostic factor in ER+ node-negative (N-) BC patients [73], even regardless of TAM therapy [74]. In addition, a recent meta-analysis has enrolled 11 BC studies, with 2958 participants, concerning the use of HOXB13/IL17BR ratio in association with a worse outcome, particularly for (N-) patients [75]. However, other validation studies have shown that even HOXB13 alone is able to predict recurrence-free survival after endocrine therapy [76,77]. Ma and collaborators have further described the molecular grade index (MGI), a five cell-cycle gene test that, in combination with HOXB13/IL17BR ratio, identifies a subgroup of early ER+BC patients with a very poor clinic outcome, despite endocrine therapy [78]. This finding suggests the identification of a new predictive test, named Breast Cancer Index (BCI), a risk index based on a combination of MGI and HOXB13/Il17BR ratio. A case-control study, performed on an independent cohort of (N-) BC patients, in order to compare the predictive value of HOXB13/IL17BR, MGI and BCI index, has shown that the three tests are associated with the risk of BC death and display a strong prognostic value in BC management [79]. Over time, BCI has become one of the most widely used predictive tests in prognostic and therapeutic stratification of patients with early-stage (N-) and lymph nodes + (L+) BC treated with TAM alone, as well as with TAM+ocretide [80].

One of the epigenetic processes responsible for the regulation of HOXB13 is related to methylation of its promoter, responsible for a reduced expression in BC cell lines. Promoter hyper-methylation of HOXB13 is more frequent in ERα+ BC patients with lymph nodes metastases. This could explain why HOXB13 up-regulation has been described in BC patients undergoing TAM therapy [81]. Furthermore, HOXB13 would confer resistance to TAM by directly regulating ERα transcription and protein expression and it is known to be able to transcriptionally up-regulate IL-6 activating the mTOR pathway [82]. An alternative mechanism related to TAM resistance may be due to HOXB13 interaction with HBXIP, an oncogenic protein promoting cancer progression. The coordinated over-expression of HOXB13 and HBXIP induces TAM resistance in ERα BC cell models: HBXIP prevents chaperone-mediated-autophagy (CMA)-dependent degradation of HOXB13 through the acetylation of its K227 residue, causing HOXB13 accumulation. HBXIP further acts as co-activator of HOXB13 to stimulate IL-6 transcription and promoting TAM resistance [83]. 

The involvement of HOXB13 in prostate gland development dates back more than 20 years and its role in PC has been soon after demonstrated [84]. Induced HOXB13 expression in PC cell models leads to cell growth inhibition with G1 cell cycle arrest linked to cyclin D1 suppression, suggesting a central role of HOXB13 as PC tumor suppressor [85]. HOXB13 interacts directly with Androgen Receptor (AR) by suppressing the hormone-mediate AR activity in a dose-responsive manner, influencing growth regulation of PC cells [86]. HOXB13 has been also investigated in androgen-independent PC, resulting over-expressed in hormone-refractory tumors. The ability of HOXB13 to modulate PC cell growth in the absence of androgen is mediated by RB-E2F signaling and inhibition of p21waf tumor suppressors [87]. The expression of HOXB13 homeoprotein by immunohistochemistry in PC patients correlates with Gleason Score (GS) and pre-operative circulating PSA levels, but no correlation with clinic-pathological features has been detected [88]. HOXB13 represents a specific biomarker of PC cells and is useful for the differential diagnosis of tumor origin, prostate versus urothelium [89], and for distinguishing metastatic tumors of prostatic origin [90]. HOXB13 has been also recently reported as sensitive and specific biomarker in pleomorphic giant cell prostate adenocarcinoma [91]. On a case series of 12400 PC samples, the aberrant expression of HOXB13 is associated with pT stage, high GS, lymph node metastases, AR expression, high pre-operative PSA level and genetic alterations, such as PTEN deletion and TMPRSS2:ERG translocation [92]. A high expression of HOXB13, AR and PSA identifies a subset of patients with a worse PC prognosis. Combined analyses of HOXB13 and PSA, in metastatic PCs, shows that only HOXB13 is able to distinguish metastatic PCs with high sensitivity and specificity [92]. HOXB13 is able to interact with different molecular pathways during PC evolution: (i) suppresses Prostate Derived ETS Factor (PDEF) [93]; (ii) suppresses p21 in castration-resistant PC, stressing this important step in PC cell survival under no androgen-influence [94]; (iii) promotes PC cell invasion and metastasis by decreasing intracellular zinc levels, enhancing NF-kB [95]. Moreover, a direct biochemical and functional interaction has been described between HOXB13 and MEIS1 in PC cells: the corresponding two proteins are co-expressed on PC tissues with the consequence of modulating PC tumor progression by prolonging HOXB13 half-life [96].

The most numerous data in literature are related to identification of germline mutations in HOXB13 sequences strongly associated with hereditary PC. The same non-synonymous mutation, a change of guanine to adenosine (c251G—A) in the second position of codon 84 (GGA—GAA), resulting in a substitution of glycine for glutamic acid (G84E), has been observed in four families with PC subjects [97]. Several population studies have associated HOXB13 G84E variant to PC risk, especially in Europe [98,99], while in Asia, in addition to G84E, another mutation (G135E) would seem to be prevalent [100]. A study designed to analyze the distribution of the variant by ethnicity, has highlighted that G84E HOXB13 is more frequent among PC patients of European decent [101]. In order to validate the value of G84E variant in clinic PC management, 2443 PC families have been recruited by the International Consortium for Prostate Cancer Genetics (ICPCG) to genotype the mutation. This study has shown that HOXB13 G84E is present in approximately 5% of PC families, mainly of European descent, confirming its association with PC risk [102]. Other studies have shown that G84E variant does not specifically characterize men with a family history of PC, but it is rather strongly associated with PC in the general population [103]. The pathogenic mechanisms associated with PC patients with G84E variant, displayed that they are characterized by the alteration of specific molecular pathways, in particular a low prevalence of better documented EGR pathways and an increased prevalence of SPINK1 pathway [104]. Several other rare missense mutations of HOXB13 gene associated with a predisposition to PC have been identified (Y88D, L144P, G216C, R229G) [97]. In addition, the analyses of the entire HOXB13 gene in 462 Portuguese familial PC subjects have revealed the presence of two novel germline mutations, supporting the concept that different rare HOXB13 mutations could be found in different ethnic groups [105]. 

HOXB13 appears to be down-regulated in about 60% of colorectal cancers (CRC). In CRC cell models, HOXB13 down-regulates the expression of T-cell-factor 4 (TCF4) and its target c-myc, inhibiting β−catenin/TCF mediated signaling. The induced expression of HOXB13 leads to the suppression of cell growth in CRC cells [106]. HOXB13 is strongly methylated in CRC cells and inhibits growth and clonogenic survival in vitro as well as in nude mice [107]. However, a recent study has shown the aberrant expression of HOXB13 in proximal colon cancers and a strong relation with lncRNA HOTAIR deregulation [108]. G84E HOXB13 mutation is very frequent also in CRC patients, suggesting an association of G84E variant with CRC risk, as occur in PC [109]. 

Abnormal HOXB13 expression has been detected in uro-genital cancers. Ovarian, cervical and endometrial cancers display HOXB13 over-expression promoting EMT, cell invasion and tumor progression [110,111,112]. In bladder cancer, HOXB13 is able to discriminate between non-muscle and muscle invasive transitional BC and its cytoplasmic de-localization represents an important prognostic value in BC patients [113]. In renal cancer HOXB13 acts as a tumor suppressor and its methylation status positively correlates with tumor grade and micro-vessels invasion [114]. The role of HOXB13 tumor suppressor has been also demonstrated in gastric cancer [114] in which HOXB13 mRNA is significantly lower in primary tumors and its de-regulation is associated with a poorer differentiation, lymph node metastases, invasion and TNM stage. HOX B13 expression is increased by the treatment of GC cells with DNA methyltransferase inhibitor 5-aza-dC[115]. In HCC patients, aberrant expression of HOXB13 is strongly associated with clinic-pathological features, such as vascular invasion, tumor grade, TNM stage and a poorer survival. In addition, it is significantly correlated with VEGF expression and microvessels density, suggesting a central role of HOXB13 in HCC angiogenesis and tumor progression [116]. Finally, an altered HOXB13 expression has been described in oral cancers [117,118] and glioma patients [119].

HOXB13 appears to be under the control of a co-localizing lncRNA. HOXB13-AS1 is a 564 nucleotide- lncRNA localized on chromosome 17, adjacent to HOXB13 [120], highly expressed in several normal human tissues [121,122] and different tumor types [119,122]. HOXB13-AS1 is able to contribute to cancer cells proliferation by binding with the enhancer of zeste homolog 2 (EZH2), epigenetically suppressing HOXB13 expression of its neighbor gene [119].

## 4. HOXC13

During embryonal development, HOXC13 is involved in the formation of skin epithelia [123] and hair follicle generation [124]. HOXC13 is an important regulator of human keratin gene expression in early trichocyte differentiation [125,126,127,128]. Aberrant HOXC13 expression has been reported in pilomatricoma [129] together with a strong expression of K5, K14 and K17 cytokeratin [130]. It is worth noting that the genes of C HOX locus are in physical contiguity to one of the two clusters of keratin genes included in the human genome [131]. Over-expression of HOXC13 in transgenic mice is also able to develop alopecia and progressive pathological skin conditions [132]. 

The involvement of HOXC13 in cell cycle progression, cell growth and carcinogenesis has been well documented: knocking down HOXC13 in human cancer cell lines, such as colorectal, breast, prostate and cervical cancer, significantly affects the viability of cancer cells [133]. HOXC13 silencing further induces cell death leading to cell cycle arrest, at G0/G1 phase, and increasing apoptosis. Finally, HOXC13-induced-expression leads to 3D-colony-formation in soft agar, highlighting its role in cell proliferation and invasion [133]. In human metastatic melanoma cell lines, HOXC13 deregulation, along with the other C HOX genes of the posterior locus, is strongly related to the expression of IL-1α, IL-6, TNFα, VLA-2, VLA-5 and VLA-6 integrins and N-RAS mutation [134]. Maeda and collaborators have shown that expression levels of HOXC13 is are higher in nevi and pT1/pT2 than in patients with pT4 melanoma, decreasing in more advanced tumors [135]. However, this data appear to be in contrast with another study in which a series of human biological samples (tissues and cells), representative of malignant melanoma progression, display that HOX C13 expression significantly increases in metastases compared to primary tumors [19]. It is well documented the ability of posterior HOX gene to generate fusion transcripts with the nucleoporin NUP98. The chimera protein NUP98/HOXC13 has a pathogenic importance in acute myeloid leukemia (AML), which leads to the deletion of the mutual fusion of the gene [136,137,138]. Subsequent molecular analyses have highlighted the fusion in frame of exon 16 of NUP98 with exon2 of HOXC13. This translocation appears to coexist with an internal tandem duplication of the gene FLT3 (fms-related tyrosine-kinase 3). Both events are crucial for the leukemiogenesis process [139]. Rare cases of AML with NUP98 rearrangements without HOXC13 involvement have also been described [140]. Aberrant HOXC13 expression has also been reported in murine models of erythroleukemia (MEL). HOXC13 binds to ETS domain of the gene PU.1 enhancing its transactional activity. The induced expression of HOXC13 and PU.1 in MEL inhibits cell differentiation suggesting a primary role in tumor cell differentiation [141]. HOXC13 is highly expressed in ameloblastoma tissues compared to keratocystic odontogenic tumors and normal mucosa [142]. Furthermore, the whole HOXC locus of the HOX network appears to be deregulated in ameloblastoma together with the keratin genes that co-localize in the same 12q13.13 chromosomal area [143]. A large study enrolling different odontogenic tumors, such as ameloblastomas, calcifying cystic odontogenic tumors, ameloblastic fibromas, keratocystic odontogenic tumors and epithelial odontogenic tumors displayed an over-expression of HOXC13 in all lesions except in fibromas [144]. HOXC13 is also over-expressed in OSCC cell models in association with altered activity of the Polycomb Repressive Complex (PCR) responsible for epigenetic modifications [145]. DNA methylation and histones alterations are strongly associated with HOX gene expression in OSCC models [146]. HOXC13 is significantly up-regulated in ESCC in association with poorer clinic-pathological features and worse prognosis of the patients, and its knockdown decreases cell proliferation and induces apoptosis in ESCC cells [147]. In cervical cancer cell model, BMI-1, a gene which encodes a ring finger protein that is the major component of the polycomb group complex 1 (PRC1), is able to modulate HOXC13 expression. The knockdown of BMI-1 in this model leads to an over-expression of HOXC13 inducing cell-cycle arrest [148]. IHC HOXC13 over-expression has also been detected in well-differentiated and de-differentiated liposarcoma tissues, in which it is strongly related to 12q13-15 chromosomal amplification [149]. A four gene signature, including HOXC13, has been proposed in PC patients, to discriminate between recurrent versus non recurrent PC and to predict the outcome of the disease [150]. HOXC13 is significantly higher in tissues and cell lines of lung adenocarcinoma, in correlation with clinic-pathological features and poorer prognosis. Knockdown of HOXC13 in lung cancer cell models inhibits cell proliferation blocking G1 phase of cell-cycle [151]. HOXC13 is further down-regulated by miR141 in lung cancer cell lines [151]. Finally, it has been recently shown that HOXC13 is over-expressed in proximal colon cancer, which strongly correlates with lymph nodes metastases and aberrant expression of lncRNA HOTAIR [108]. 

The 5’ end *HOXC* region contains several lncRNAs, including HOXC13-AS (adjacent to HOXC13), HOXC-AS2, HOXC-AS3 transcripts [152], and HOTAIR. However, none of them plays a role in the interaction/regulation of HOXC13, both in normal and pathological conditions. HOXC13-AS is highly expressed in head and neck squamous carcinoma (HNSC) tissues and its aberrant expression is detectable in nasopharyngeal carcinoma (NPC) tissues and cell line. Knockdown of HOXC13-AS leads to an increase of NPC cell proliferation, migration and invasion [153].

LncRNA HOX Transcript Antisense Intergenic RNA (HOTAIR) located on chr.12q13.13 (between HOXC11 and HOXC12) is able to transcriptionally repress *in trans* the 5’ end of HOXD locus on chr. 2q32-33. [154,155]. HOTAIR acts as a regulator of chromatin states by binding PRC2, with its 5’end. HOTAIR further interacts, through its 3’ end, with LSD1 (lysine-specific demethylase 1), a central player in epigenetic regulation. HOTAIR is able to: (i) promote the epigenetic activation/repression of gene expression; (ii) affect the target suppression of gene expression through competitive binding to miRNAs; (iii) modify gene expression, at post-transcriptional level, interacting with transcription factors and ribosomes or binding to splicing factors [15]. Aberrant HOTAIR expression has been detected in several human cancers associating its role with tumor proliferation, angiogenesis, progression, drug resistance and worse prognosis [156]. In addition, numerous experimental evidences have focused the attention on the potential role of HOTAIR as circulating marker in cancer patients and as potential therapeutic target [157]. A potential relation between HOXC13 and HOTAIR expression has been shown only in a recent study on colon cancer in which both genes are co-expressed in the right CRCs samples and are correlated with lymph nodes metastases [108].

## 5. HOXD13

During development, HOXD13 plays a central role in the formation of the limbs [158,159], in a part of the gut [160] and in genitourinary system function [25]. Mutations in its structure (expansions of a polyalanine stretch in the amino-terminal region) are able to generate synpolydactyly, an inherited human abnormality of the hands and feet [161].

HOXD13 deregulation in human cancers mostly concerns haematological malignancies, less frequently than in other types of tumor. HOXD13, as the other posterior HOX genes, is involved in a chromosomal translocation in AML with the generation of a chimeric protein between HOXD13 and NUP98 [162]. The analyses of the fusion gene, in a murine hematopoietic model, show its involvement in the growth and differentiation of early hematopoietic progenitor cells. In addition, co-transduction of NUP98-HOXD13 transcript, plus Meis1 cofactor, induces lethal AML in mice models, highlighting their fundamental role in leukemic transformation [163]. During leukemic evolution, NUP98-HOXD13 interacts, besides Meis1, with MN1, a transcriptional co-activator forming fusion transcripts with TEL, GATA2, ERG, Epor and miR291/miR29b1 genes [164]. However, co-transduction of an activated NUP98-HOXD13 fusion gene and MN1 alone is able to induce AML in engrafted mice [165]. More recently, it has been demonstrated that the loss of Toll-like receptor 2 too is able to accelerate leukemic progression in NUP98-HOXD13 mouse model [166]. During leukemic progression, the presence of the fusion transcript NUP98-HOXD13 is associated with other hematopoietic disorders, such as myelodysplastic syndrome (MDS), chronic myeloid leukemia and blast crisis. Transgenic mice, expressing the fusion transcript, develop MDS; more than half of them progress to acute leukemia or display megakaryocytic differentiation and increase bone marrow apoptosis [167,168]. Other molecular alterations are associated with NUP98-HOXD13 in AML transformation. FLT3/ITD mutation is involved with leukemic transformation: its insertion in murine model induces only an MDS phenotype. In contrast, the co-expression of FLT3/ITD and NUP98-HOXD13, in the same model, induces AML with 100% penetration and short latency [169]. Furthermore, the loss of p15lnk4b together with NUP98-HOXD13 trans-gene leads to the development of Myeloid neoplasia, AML, Myelo-proliferative disease and MDS [170]. NUP98-HOXD13 fusion gene occurs also in non-lymphocytic-leukemia and it is coupled with NRAS, KRAS and Cbl gene mutations, in transgenic mice model [171]. Moreover, NUP98-HOXD13 drives the loss of one or both p53 alleles strengthening MDS phenotype and accelerating acute myeloid leukemia development [172].

A large IHC study performed on 4000 normal and neoplastic tissue samples, which included 79 different tumor categories, has shown that the over-expression of HOXD13 is prevalent in cancer tissues, compared to non-neoplastic samples, particularly in breast, colon and salivary glands cancers. However, in several tumor types, such as pancreatic and gastric cancers, HOXD13 has displayed the opposite trend being strongly down-regulated, which suggests also for this gene a dual role during tumor evolution, as oncogene and tumor suppressor [29]. Deregulation of HOXD13 displays a prognostic value in breast cancer. HOXD13 down-regulation is significantly associated with tumor size, lymph-node metastases and a poorer overall survival [173]. HOXD13 expression has been further proved to be a useful diagnostic tool in BC in combination with magnetic resonance imaging (MRI) [174]. HOXD13 promoter is methylated in about 60% of BC and the methylation status strongly correlates with clinic-pathological characteristics and a poorer survival of BC patients [175]. Moreover, the detection of HOXD13 methylation status in circulating free DNA (cfDNA) from serum, has proved to be a useful tool for BC patients management [176]. HOXD13 methylation status is associated with lung adenocarcinoma prognosis, suggesting, also for this tumor, its diagnostic value [177]. A microarray analysis reveals that posterior HOXD genes are involved in bone formation and hyper-expressed in primary Ewing sarcoma (ES). While posterior HOXD genes (from HOXD13 to HOXD9) promote chondrogenic differentiation and enhance bone-associated gene expression, HOXD11 and HOXD13 are specifically involved in cell growth and invasion of Ewing sarcoma. Their knockdown significantly suppresses lung metastases in mice models, suggesting a role in the metastatic potential of ES cells [178]. A cytogenetic analysis reveals the frequent chromosome copy gain at chr. 2q24, a region centromeric to HOXD13, in hepatoblastoma patients. Moreover, 2q24 gain is an independent factor able to predict poor outcome, suggesting the presence in this chromosomal area of a tumor suppressor gene involved in hepatoblastoma evolution [179].

The posterior HOXD locus, in addition to being regulated in *trans* by lncRNA *HOTAIR,* is under the control of two more lncRNAs, named Hotdog (HOG) and Twin of Hotdog (*TOG*), which are located in a desert zone of the gene adjacent to HOXD13 gene [180]. HOG/TOG is critical in the regulation of HOXD genes during the caecum development [180] but there is no information on its role during tumor transformation and progression. 

## 6. Conclusions

**Figure 1:**
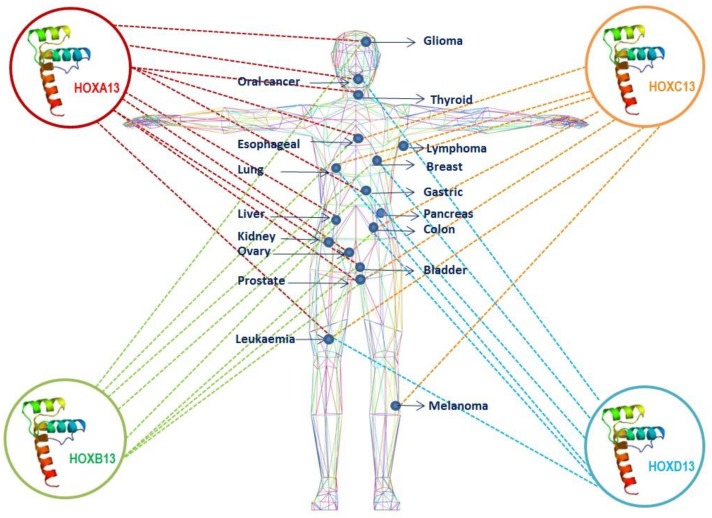
Schematic representation of the paralogous HOX13 genes involvement in the main human cancers.

The aim of this review was to summarize and comment on the numerous experimental evidences on the fundamental role of the most posterior genes of the HOX gene network in cancer diseases. Although the whole HOX gene network acts in a coordinated manner in body plan organization during development and in the maintenance of the phenotypic identity in human adult tissues and organs, deregulation of HOX13 paralogues has been strongly related to severe alterations of body structures [30,160] and associated with different molecular pathways promoting tumor diseases [32,181,182] (Figure 1). Since deregulation of HOX13 paralogues genes has been widely associated with invasion/metastasis [19,88] and drug resistance processes [83], their role has been suggested both as tumor diagnostic markers and as prognostic-predictive markers. 

Even if HOX genes came from a common ancestral HOX gene, some of them appear to play different roles during cancer development and progression. In most human cancers, HOXB13 would seem to behave as a tumor suppressor gene, while for the other HOX13 paralogues genes a role as oncogenes is mainly described. HOX proteins act as transcription factors, therefore this remarkable functional difference may be due to their ability to interact with different target genes modulating many molecular pathways involved in proliferation, migration, and invasion. Further functional studies should be carried out to better define their mechanisms of action in order to modulate both their activation and inhibition. Blocking of HOX proteins activity by interfering with their binding to PBX co-factor, has been able to reduce tumor cell growth and induce apoptosis, supporting the therapeutic potential of inhibiting HOX/PBX dimer formation in cancer [183,184,185]. 

The ability to detect HOX13 proteins, as well as lncRNAs with which they co-localize/interact, in biological fluids [186,187,188], combined with therapeutic strategies that interfere with their activity, would open a new scenario in the management of cancer patients.
